# Improving Health for Older Adults With Pain Through Engagement: Protocol for Tailoring and Open Pilot Testing of a Mind-Body Activity Program Delivered Within Shared Medical Visits in an Underserved Community Clinic

**DOI:** 10.2196/52117

**Published:** 2023-12-29

**Authors:** Katherine McDermott, Nadine Levey, Julie Brewer, Madison Ehmann, Julia E Hooker, Roger Pasinski, Neda Yousif, Vidya Raju, Milton Gholston, Jonathan Greenberg, Christine S Ritchie, Ana-Maria Vranceanu

**Affiliations:** 1 Center for Health Outcomes and Interdisciplinary Research Department of Psychiatry Massachusetts General Hospital Boston, MA United States; 2 Massachusetts General Hospital Revere HealthCare Center Revere, MA United States; 3 Center for Aging and Serious Illness Massachusetts General Hospital Boston, MA United States

**Keywords:** chronic pain, mind-body, underserved, musculoskeletal pain, pain, older adults, pain management, feasibility, intervention

## Abstract

**Background:**

Chronic musculoskeletal pain is prevalent and disabling among older adults in underserved communities. Psychosocial pain management is more effective than pharmacological treatment in older adults. However, underserved community clinics often lack psychosocial treatments, in part because of a lack of trained providers. Shared medical appointments, in which patients undergo brief medical evaluation, monitoring, counseling, and group support, are an efficacious and cost-effective method for chronic disease management in underserved clinics, reducing the need for specialized providers. However, shared medical visits are often ineffective for chronic pain, possibly owing to lack of inclusion of skills most relevant for older adults (eg, pacing to increase engagement in daily activities).

**Objective:**

We have described the protocol for the development and initial pilot effectiveness testing of the *GetActive+* mind-body activity intervention for older adults with chronic pain. *GetActive+* was adapted from GetActive, an evidence-based intervention that improved pain outcomes among mostly affluent White adults. We aim to establish the initial feasibility, acceptability, fidelity, and effectiveness of *GetActive+* when delivered as part of shared medical appointments in a community clinic.

**Methods:**

We conducted qualitative focus groups and individual interviews with providers (n=25) and English-speaking older adults (aged ≥55 y; n=18) with chronic pain to understand the pain experience in this population, perceptions about intervention content, and barriers to and facilitators of intervention participation and implementation in this setting. Qualitative interviews with Spanish-speaking older adults are in progress and will inform a future open pilot of the intervention in Spanish. We are currently conducting an open pilot study with exit interviews in English (n=30 individuals in total). Primary outcomes are feasibility (≥75% of patients who are approached agree to participate), acceptability (≥75% of patients who enrolled complete 8 out of 10 sessions; qualitative), and fidelity (≥75% of session components are delivered as intended). Secondary outcomes include physical function—self-reported, performance based (6-minute walk test), and objective (step count)—and emotional function (depression and anxiety). Other assessments include putative mechanisms (eg, mindfulness and pain catastrophizing).

**Results:**

We began enrolling participants for the qualitative phase in November 2022 and the open pilot phase in May 2023. We completed the qualitative phase with providers and English-speaking patients, and the results are being analyzed using a hybrid, inductive-deductive approach. We conducted rapid analysis of these data to develop *GetActive+* before the open pilot in English, including increasing readability and clarity of language, reducing the number of skills taught to increase time for individual check-ins and group participation, and increasing experiential exercises for skill uptake.

**Conclusions:**

We provide a blueprint for the refinement of a mind-body activity intervention for older adults with chronic pain in underserved community clinics and for incorporation within shared medical visits. It will inform a future, fully powered, effectiveness-implementation trial of *GetActive+* to help address the chronic pain epidemic among older adults.

**Trial Registration:**

ClinicalTrials.gov NCT05782231; https://clinicaltrials.gov/study/NCT05782231

**International Registered Report Identifier (IRRID):**

DERR1-10.2196/52117

## Introduction

### Background

Chronic musculoskeletal pain (pain that persists for >3 mo [1]) is highly prevalent among older adults and is associated with substantial clinical, economic, and societal burden [2-4]. Chronic pain tends to be more complex in older adults, with approximately 60% to 70% describing pain in multiple sites alongside multiple comorbidities [5]. Among older adults, chronic pain leads to significant decline in physical and emotional function, with substantial disability from reduced mobility, avoidance of activity, falls, depression, anxiety, sleep impairment, and social isolation [2,3,6,7] regardless of the location and severity of pain [8,9]. Sedentariness, lack of engagement in activities of daily living, and impairments in balance and gait [10] are common in older adults with chronic pain and further increase the risk for morbidity and mortality [2].

For older adults, pain medications, including opioid analgesics, have limited efficacy [11,12], increase the risk for adverse events such as falls [13], and can lead to confusion and cognitive decline [14]. Nonpharmacological treatments for chronic pain such as cognitive behavioral therapy or mindfulness-based interventions are safe for older adults and can improve pain outcomes including physical and emotional function [15,16]. However, access to timely treatment is scarce [17], and treatments often fail to target physical function components that are critical in older adults, including walking and activities of daily living [18].

Importantly, treatment access is not evenly distributed. Older adults from communities of high socioeconomic status have substantially greater likelihood of receiving psychosocial pain management, whereas individuals from underserved communities often lack psychosocial treatment options, despite being at the highest risk for negative pain outcomes [19,20]. Disparities in chronic pain management are multidimensional, including divisions in patient and health care provider communication, variability in decision-making, and gaps in access to effective treatment [19,21]. Furthermore, older adults from underserved communities receive most of their care from primary care practitioners. However, many primary care practitioners do not have the time or training to provide psychosocial pain management and tend to primarily focus on pharmacological treatment. Access to nonpharmacological therapies is limited for many older adults from disadvantaged populations because therapies are not affordable, not recommended by providers, or not available in their community [22-25].

There is a need to implement psychosocial pain management interventions within primary care that are accessible to underserved older adults with chronic pain. Shared medical visits may be a viable model to overcome the many barriers to implementation in these communities [26,27]. Shared medical visits seek to improve patient health through a blend of medical care, education, and peer support [28]. They include group conversations, individual check-ins, and peer interactions as key elements [29]. Shared medical visits are often used to provide greater access to complementary and integrative health approaches to pain management, including mindfulness-based interventions [30]. Patients report that shared medical visits allow them to learn from peers’ experiences and create a sense of connection [31,32], with many preferring them to individual primary care appointments [33]. There is evidence to suggest that pain management can be delivered successfully in shared medical visits [34,35], including with economically and ethnically diverse populations [36-38]. Patients qualitatively support shared medical visits and demonstrate increases in quantitative measures of quality of life [33,35,39]. However, pain outcomes, including pain interference and pain catastrophizing, are often not adequately targeted by existing shared medical visit interventions [34,39]. Existing interventions are often broad in their scope, including using mindfulness, guided imagery, health behaviors (eg, exercise and nutrition education), yoga, chiropractic education, and acupuncture [33,35,37,39], without enough attention to skill uptake and integration into daily life. Although experiential exercises are sometimes incorporated, less time is allocated for repetition and troubleshooting skills use to acquire mastery. Furthermore, many of these programs were developed for young populations; there is an absence of skills focused directly on activities of daily living, which is particularly important to older adults [40].

### Objective

To address the need for accessible, psychosocial, group pain management for older adults, we are iteratively adapting our evidence-based chronic pain intervention, the GetActive program [41], for implementation in an underserved community clinic using shared medical visits performed in English and Spanish. GetActive uses cognitive behavioral therapy (eg, identifying and challenging negative thoughts) and mind-body skills (eg, deep breathing and mindfulness) to promote engagement in physical activity [42]. Our team has previously reported on the development and testing of GetActive including (1) strong feasibility, acceptability, and credibility [43,44]; (2) moderate to large significant improvements in self-reported physical function, performance-based physical function (6-minute walk test; 6MWT), pain, and emotional function (depression and anxiety) [43,45]; and (3) improvements through the mechanistic intervention targets (pain catastrophizing, fear of pain, pain resiliency, mindfulness, coping, and self-compassion) [44,46-48]. However, the original *>GetActive* program was developed in a predominantly White and affluent sample and did not use the shared medical visit format.

Our new intervention, *GetActive+*, will be delivered within the shared medical visit format. Each group session will include both pain management skills and a medical check-in with a nurse practitioner, to monitor fall risk and ability to engage in the physical activities that form the cornerstone of the program. Our approach to developing *GetActive+* is informed by the socioecological model [49], Aarons’ stage model [50], and Proctor’s framework for implementation [51]. Development will be iterative and will entail (1) using qualitative methods (focus groups and individual interviews) to assess provider and patient perceptions about the program and treatment needs and (2) conducting an open pilot with qualitative exit interviews to examine feasibility, acceptability, fidelity, and preliminary effectiveness in improving physical and emotional function and putative mechanisms. We will use qualitative information from the exit interviews to triangulate acceptability and obtain patient feedback about the treatment components and study procedures. We hypothesize that *GetActive+* will be feasible (≥75% agree to participate), be acceptable (≥75% of those enrolled complete 8 out of 10 sessions), be delivered with fidelity (*≥*75% sessions are delivered as intended), and show evidence for effectiveness in improving quantitative outcomes. We will use the information gained from the open pilot to refine the intervention and finalize the manual, assessments, and study protocol to support the successful completion of a future, hybrid, type-1, effectiveness-implementation trial. As a large portion of older adults in community clinics speak Spanish, we are also developing a Spanish version of *GetActive+*. Our ultimate goal is to create a program that can be delivered by providers of any specialty in a group format, to enhance the feasibility of dissemination to any community clinic. Given that different clinics have different resources, provider availability, and patient payment systems, this flexibility will maximize patient access while minimizing copayments.

In this paper, we have described the protocol and status of the qualitative phase and open pilot of *GetActive+*. We have discussed the challenges and flexibility required to refine and adapt a manualized chronic pain treatment for this setting. Ultimately, we aim to provide a framework for increasing access to evidence-based psychosocial pain management for older adults with chronic pain in underserved communities.

## Methods

### Phase 1: Qualitative Interviews

#### Study Design

We elicited feedback from stakeholders (providers and patients) through qualitative focus groups and individual interviews, with the goal of identifying and addressing barriers to and facilitators of implementation. Interviews were delivered in person at the clinic or via secure, live videoconferencing. Staff participants included primary care physicians, nurses, physical therapists, medical interpreters, and administrative staff. Ongoing interviews with Spanish-speaking patients are conducted by trained native speakers.

#### Setting and Participants

This study was conducted at the Massachusetts General Hospital (MGH)–Revere HealthCare Center (RHC), a community health care clinic that serves an economically and ethnically diverse population, approximately half of whom identify as immigrants, Asian, Black, Latino, or multiracial.

The inclusion criterion for providers for the qualitative interviews was as follows: any staff member of RHC. The inclusion criteria for patients for the qualitative interviews were as follows: (1) a patient at RHC; (2) aged ≥55 years; (3) self-reported chronic musculoskeletal pain (ie, pain duration >3 mo); (4) pain score ≥4 on the Numerical Rating Scale [52]; (5) no self-reported cognitive challenges that would interfere with participation (eg, cognitive impairment or dementia); (6) no self-reported, currently active, untreated psychotic or substance use disorder; (7) self-reported ability to walk for at least 6 minutes, including with assistive devices; and (8) English or Spanish fluency. Exclusion criteria were as follows: (1) current serious medical illness that is expected to worsen in the next 6 months (eg, advanced cancer) and (2) currently active and untreated serious mental health condition (eg, bipolar disorder, schizophrenia, or substance use disorder) that could interfere with focus group participation. Focus groups were conducted separately with patients and providers.

#### Recruitment, Screening, and Enrollment

The RHC medical director or study champions—primary care physicians in the clinic who assisted in recruitment—identified potential participants for the qualitative phase with RHC staff. All RHC staff were notified that their participation in the focus groups or interviews was voluntary, that results would not be reported to leadership, and that they will be able to withdraw at any time. Participants reviewed the institutional review board–approved study fact sheet independently using REDCap (Research Electronic Data Capture; Vanderbilt University) tools hosted at MGH; a HIPAA (Health Insurance Portability and Accountability Act)-approved electronic data capture system) [53]. Those who indicated their verbal agreement to participate were enrolled.

Potential participants for the focus groups and interviews with RHC patients were recruited via referrals from study champions and other RHC medical providers. Study champions encouraged referrals from fellow staff. Patient participants were also recruited through flyers posted in the clinic and an advertisement on the MGH web research recruitment platform. Following eligibility screening by a research assistant, participants reviewed a study fact sheet and provided verbal consent to participate. Participants were notified that their participation in the focus groups or interviews was voluntary and that they will be able to withdraw at any time. Patients who were ineligible or uninterested were given a chronic pain resource sheet.

#### Qualitative Interviews

We used a similar semistructured, qualitative interview guide for both provider and patient interviews. Qualitative interview guides centered on three major domains: (1) needs and characteristics of older adults with chronic pain at RHC, including how environmental, sociocultural, behavioral, and medical factors influence and are influenced by pain and perceptions about pain treatments; (2) program preferences, including feedback about the general program content, structure, format, and skills (including audio examples of program skills); and (3) potential barriers to and facilitators of program participation and implementation, including questions related to transportation and homework completion. Interviews were conducted by trained facilitators, primarily, clinical psychologists and a trained research assistant. A trained bilingual research assistant conducted the Spanish focus groups and interviews.

Multiple reminder calls were conducted, and emails were sent before the focus groups. Nevertheless, on multiple occasions, attendance was not sufficient to conduct the group. Potential participants expressed that they had other competing responsibilities (eg, caregiving roles and medical appointments) that interfered with focus group participation. Therefore, we adopted a more flexible procedure, allowing for the completion of individual interviews via Zoom (Zoom Video Communications) or phone to ensure that we could get the information needed to inform program refinement while working with the needs of the clinic population.

#### Data Coding and Analysis

We conducted rapid data analysis (RDA) following each interview to determine real-time data saturation and develop *GetActive+* and the study protocol before conducting more extensive qualitative analyses [54]. RDA is a valid and reliable qualitative data analysis method that is recommended in studies where there is a need for real-time data to inform intervention adaptation and implementation [54-56]. RDA bypasses the process of transcription and in-depth coding; instead, data are organized immediately following qualitative interviews based on a template created from the interview script [57]. In this study, RDA was conducted within 24 hours of the interview, typically immediately after its completion. Our RDA template was organized into the following domains: (1) living with chronic pain: challenges, needs, and treatment (eg, description of the clinic population); (2) program implementation and preferences (eg, feedback about the core program skills); and (3) barriers to and facilitators of program implementation (eg, barriers to and facilitators of patient participation and homework completion). On the basis of the RDA literature, the completed template was reviewed by another member of the study team and entered into a matrix of responses [55,58], which informed the manual and procedure adaptations before the open pilot. The matrix was organized in a hybrid, inductive-deductive manner based on a combination of the domains from the RDA template and the information that emerged from qualitative interviews that was most useful in guiding the program adaptations. Each row consisted of one of the following domains: clinic population, usual pain care at the clinic, initial reactions to the program, format and delivery modality, core skills, program barriers and facilitators, and focus group recommendations. Each column represented a single group or individual interview.

Formal, planned, qualitative analyses are ongoing and will be used to provide an in-depth synthesis of the qualitative data, including comparisons between provider and patient feedback. These analyses involve the transcription of audio recordings and data analysis using the program, Dedoose [59]. We generated a qualitative codebook using a hybrid, deductive-inductive approach [60], wherein codes are created based on a priori categorizations using theoretical frameworks (eg, the socioecological model) and then revised based on novel content identified from the data. We allowed for new codes to be created directly from the data analysis process. Overall, 2 trained research assistants coded the first 20% of the transcripts under the guidance of a psychologist to resolve discrepancies between coders until sufficient concordance was achieved, following which the codebook was solidified, and the remaining transcripts were coded by a single coder.

#### Intervention Refinement

We used RDA to inform intervention refinement. We made the following modifications: (1) added psychoeducation about the nature of chronic pain to increase the understanding of core program principles and improve treatment buy-in; (2) simplified the program language and presentation to ensure that the manual was concise and at a sixth-grade reading level; and (3) consolidated the skills and ensured more time for experiential learning, check-ins, and group discussions to facilitate both skill mastery and social connection. In addition, because of high rates of stress, trauma, and grief in this clinic population, we focused the program skills on the impact of these factors on the pain experience and discussed how program skills can target both pain and these psychosocial factors. For example, the importance of using mindfulness to facilitate awareness about the impacts of stressors on pain was noted throughout the refined intervention. Finally, consistent with the goals of our subsequent effectiveness trial, the program emphasizes flexibility within fidelity, whereby we aim to deliver the active ingredients of the program while allowing flexibility with regard to the delivery format (eg, in-person or virtual), skill delivery contextualization, and support for the interventionist to adapt and differentiate support based on need [61]. Additional modifications and cultural tailoring of the intervention are expected as we develop the Spanish version of *GetActive+*.

### Phase 2: Open Pilot of *GetActive+* With Exit Interviews

#### Study Design

*GetActive+* is a 10-week group program designed to be led by any provider available in a community clinic. The open pilot English version of *GetActive+* is delivered by a mental health professional and a nurse practitioner in groups of 3 to 10 members. The nurse practitioner conducts 5-minute, individual, medical check-ins before or after the group session to assess current pain levels and discuss medical concerns that could interfere with activity engagement (eg, acute injuries and fall risk). The medical check-ins are optional. The nurse practitioner can refer to additional medical care, including contacting primary care providers, as needed.

The open pilot study is registered on ClinicalTrials.gov (trial registration: NCT05782231).

#### Setting and Participants

Inclusion criteria for the open pilot are identical to those for the qualitative phase, with the following exceptions: patients must be cleared for participation by medical staff and cognitive functioning is assessed using the Short Portable Mental Health Questionnaire [62] with a requirement of <4 errors to participate. An additional exclusion criterion was added that excluded those unable or unwilling to wear an ActiGraph, a device required for baseline and postintervention assessment of objective physical function.

Initial recruitment, screening, and enrollment procedures are identical to those of phase 1. Once a patient referral is received, a research assistant conducts a phone screening call with the patient. The research assistant provides a detailed introduction of the program, explaining the aim, format, modality, and participant requirements. All patient information, contacts, screening outcomes, and participant status are recorded in a secured, study-specific tracking log. If a patient is eligible and interested in participating, the research assistant collects their availability and preferred method of contact. If a patient is deemed ineligible, they are emailed a chronic pain resource sheet with information about pain management. Eligible participants are scheduled for an in-person clinic visit, where they provide written informed consent and complete the baseline assessments. Baseline sessions are scheduled based on the availability of the group leader, participants, and room to work within the clinic’s existing programming. The research assistant conducts a scheduling call to all participants, encouraging each individual to record the date and time of the baseline session in their phone, in their calendar, or on their refrigerator. Owing to our issues with focus group attendance, as documented previously, we instituted a system of reminder calls to ensure attendance. Participants receive one call in the week before the baseline session and another on the day of the baseline session. Once the program begins, participants receive 1 reminder call in the morning of the session. They additionally receive calls if there are missing assessments or if follow-up information is needed regarding a concern that arose in the group (eg, safety considerations after a fall). We use a flexible approach in adapting to the needs of the group members, including providing GrandPad tablets for older adults with low income, giving them secure access to the study website (including skill recordings), assessments, and Zoom (for those who attend virtually).

#### Intervention

*GetActive+* session contents are described in Table 1. The program begins with pain education, including information about how pain originates in the brain rather than because of tissue damage, that any stressors that dysregulate the nervous system can increase pain, and that chronic pain often involves a false alarm that fires in the absence of actual ongoing harm to the muscles and tissues. This information is presented to reinforce that participation in activities of daily living including light activity (eg, walking) is safe, regardless of the presence of pain. Participants are informed that the purpose of the program is to increase the time they spend in being active, such as engaging in walking, active hobbies, or chores. They are asked to reflect about their typical day and to contemplate what their day would look like if the program was successful and how they would like to increase their activity in ways that are meaningful to them. *GetActive+* then focuses on a series of skills in 4 categories, with primary focus on activity engagement skills including participating in active chores and hobbies that are meaningful and quota-based pacing to safely break the connection between pain and activity. Quota-based pacing is the cornerstone of the intervention and consists of setting weekly, realistic activity goals to increase participants’ engagement in active hobbies, chores, and walking over time (eg, increasing activity by 5-10 min/d each week). The purpose of quota-based pacing is to build a sustainable habit that can be continued after the program ends. Emphasis is placed on the individuality of the participant’s experience such that they are encouraged to choose activities that are meaningful to them. Similarly, they are provided with a range of complementary skills throughout the program to assist them in increasing activity and reducing pain interference; they are encouraged to choose those that are most applicable to their individual goals and experience. These skills are mind-body skills (eg, deep breathing, body scan, mindful walking, self-compassion, social support, and acceptance) to change one’s relationship with pain (eg, reduce reactivity, fear, and ruminative self-talk through relaxation response and mindfulness exercises) and to facilitate activity engagement, pain behavior awareness skills to understand the “downward spiral” (eg, how lack of activity perpetuates chronic pain and emotional and physical disfunction), and cognitive skills to learn to identify and challenge negative automatic thoughts. All participants receive a treatment manual with descriptions of the skills and worksheets to record their individual cognitions, emotions, and behaviors surrounding pain and their weekly pacing plans to increase activity engagement.

**Table 1:** *GetActive+* intervention content in each session.

Session	Topic	Skills
1	Chronic pain education	Identifying the true vs false pain alarms and understanding the downward spiral of pain disability
2	Catching unhelpful thoughts and increasing activity	Identifying negative automatic thoughts that lead to pain interference and introducing quota-based pacing
3	Becoming aware	Mindfulness and body scan
4	Staying engaged with life	Mindful walking and engagement in meaningful activities
5	Working with unhelpful thoughts	Education about cognitive distortions and challenging negative automatic thoughts
6	Taking a pause to check in	Review of all skills, progress in activity engagement, and motivation to continue
7	Caring for yourself when you are in pain	Mindfulness of pain and self-compassion
8	Feeling connected with others	Discussion about the impact of pain on social connection and how to increase connection, including through walking with others
9	Promoting acceptance	Acceptance, including finger trap demonstration
10	Staying on track and maintaining your progress	Review of skills and plan for continuing engagement in activity following treatment

The beginning of each session consists of an overview and check-in regarding activity goals and skill use. Depth rather than breadth is emphasized in the check-in, in that clinicians are instructed to troubleshoot a particular skill, activity goal, or barrier to increasing activity, rather than reviewing the week in its entirety. Clinicians form a case conceptualization to individualize the intervention and target participant-specific barriers to activity engagement. For example, a participant with increased anxiety might be encouraged to use mindfulness skills to notice how fears about pain are preventing them from engaging in activity, whereas a participant who tends toward stoically increasing activity regardless of consequences would be encouraged to use mindfulness skills to notice when they are entering a boom-and-bust cycle. Participants are encouraged to try all the skills and are informed that skills are often most helpful with practice; however, it is also noted that the program represents a toolkit of skills and that, ultimately, they can choose those they feel are most applicable to them. We anticipate that the *GetActive+* Spanish version will retain the core components of the program, with additional tailoring based on information from qualitative interviews and focus groups.

Clinicians note the feedback about the intervention components following each session, including whether skills are understood, require additional clarification, and are perceived as useful. This information will be used to further refine *GetActive+* at the end of the open pilot phase.

#### Treatment Fidelity

Consistent with our ultimate goal of creating a skills-based activity program that can be flexible and delivered by any provider, we are using interdisciplinary clinician teams. English-speaking groups are currently led by a Doctor of Philosophy–level clinical psychologist in conjunction with a nurse practitioner. The psychologist has experience with both chronic pain management and skill training intervention groups. The nurse practitioner is experienced in geriatrics, including pain management and medical considerations of older adult populations. In the open pilot, we used a sequential approach to training in which the nurse practitioner completed training on the program, consisting of a description of the development and initial testing of GetActive, theoretical rationale and core principles of the program, core skills, and role plays. She observed 1 group led by the clinical psychologist, codelivered the second group, and is currently leading the delivery of the third group, which will be recorded and rated for competency. She will be rated on (1) *GetActive+* general intervention principles and theoretical background; (2) *GetActive+* skill delivery; and (3) nonspecific competencies such as establishing rapport with participants, problem solving, and time management. The lead clinician, in conjunction with the study team, created a clinician version of the manual, informed by the experience of conducting the intervention in the open pilot. The clinician manual consists of guidance about timing, suggested language, and advice about how to maximize the key targets of the intervention. We opted to have the open pilot sessions cofacilitated by a mental health professional (psychologist and social worker) and nurse practitioner together as a way to allow the nurse practitioner to contribute to the clinician manual with real-time feedback. This manual will allow for wide dissemination, so that any provider, regardless of background, will be able to deliver the intervention.

#### Intervention Fidelity

We use session content adherence checklists to ensure that all components of the intervention are delivered in compliance with the study protocol. Clinicians complete an adherence checklist and a session note summarizing the session content and any issues that arise regarding individual concerns and progress and any information that is important for case conceptualization. Sessions are recorded, and 20% (6/30) will be reviewed for fidelity. Feedback is provided during weekly supervision, led by one of the study’s principal investigators (A-MV). Participants who miss a session are offered one-on-one makeup sessions. Home practice is logged manually by participants, tracked through the completion of web-based surveys sent either through email or SMS text message, and discussed in each session by the clinicians. Issues with compliance are discussed in supervision. Procedures follow the National Institutes of Health (NIH) Science of Behavior Change recommendations [63] and have been used by the multiple principal investigators and in previous clinical trials.

#### Assessments

##### Overview

**Figure 1:**
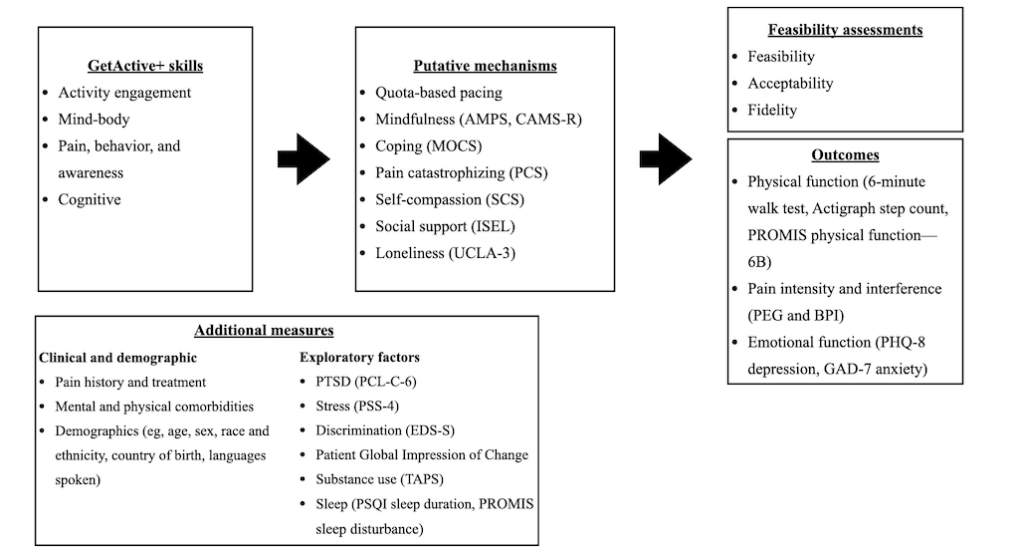
Diagram of *GetActive+* skills and assessments. AMPS: Applied Mindfulness Process Scale; BPI: Brief Pain Inventory; CAMS-R: Cognitive and Affective Mindfulness Scale–Revised; EDS-S: Everyday Discrimination Scale–Short; GAD-7: 7-item Generalized Anxiety Disorder Scale; ISEL: Interpersonal Support Evaluation List; MOCS: Measure of Current Status; PCL-C-6: 6-item Post-Traumatic Checklist–Civilian Version; PCS: Pain Catastrophizing Scale; PEG: Pain, Enjoyment of Life, and General Activity scale; PHQ-8: 8-item Patient Health Questionnaire; PROMIS: Patient-Reported Outcomes Measurement Information System; PSQI: Pittsburgh Sleep Quality Index; PSS-4: 4-item Perceived Stress Scale; PTSD: posttraumatic stress disorder; SCS: Self-Compassion Scale; TAPS: Tobacco, Alcohol, Prescription Medications, and Other Substance; UCLA-3: University of California Los Angeles–3 Loneliness Scale.

Study assessments are depicted in Figure 1. Self-report measures and behavioral assessments of physical function (6MWT and ActiGraph-measured step count) are administered at baseline (1 week before session 1) and after the intervention (1 wk following session 10). Measures are collected either electronically (using the secure, HIPAA-approved REDCap platform on a study device) or using pen and paper, depending on device availability and participant preference. Measures are administered before and after the intervention, with the exception of the demographic questions (baseline only) and Patient Global Impression of Change (after the intervention only).

##### Primary Outcomes

Primary outcomes for the open pilot feasibility trial are described in Table 2 and include a priori benchmarks for feasibility, acceptability, and fidelity, informed by Aarons’ stage model [50] and Proctor’s framework for implementation [51].

**Table 2:** Feasibility benchmarks.

Implementation outcome	Definition	Recommended implementation step in the model by Aaron et al [50]	Benchmark
Feasibility	Suitability and practicality	Early (steps 1-3)	≥75% of those who are approached agree to participate in intervention; qualitative feedback
Acceptability	Satisfaction with or tolerability of the proposed approach	Early (steps 1-3)	≥75% of those who are enrolled complete at least 8 out of 10 sessions; qualitative feedback
Fidelity	Delivery of *GetActive+* as intended	Early to middle (steps 2-3)	≥75% of the session components are delivered by clinicians as intended; 20% of the sessions are rated

##### Secondary Outcomes

We will include the following: (1) performance-based, objective, and self-reported physical function; (2) pain intensity and interference; (3) depression symptoms; and (4) anxiety symptoms.

Performance-based physical function is measured using 6MWT [64,65]. 6MWT is designed to measure the total distance covered by an individual in 6 minutes, with greater distances covered indicating better physical function. Objective physical function is assessed using ActiGraph GT3X-BTLE [66], which measures average step count over the course of a 5-day to 7-day period. Self-reported physical function is assessed using the Patient-Reported Outcomes Measurement Information System Physical Functioning Short Form 6b [67], a measure of the ability to engage in physical activities, including active household tasks. Scores range from 8 to 40, with higher scores indicating greater physical function. Self-reported physical function will be the primary outcome in the subsequent fully powered trial.

We assess pain intensity and interference using the Brief Pain Inventory–Short Form [68]. The pain severity subscale measures pain at its worst, least, average, and current state. The pain interference subscale assesses interference of pain in general activity, mood, walking ability, work, relationships, sleep, and enjoyment of life. Scores for both subscales range from 0 to 10, with higher scores indicating more severe pain severity or interference. Depressive symptoms are assessed using the 8-item Patient Health Questionnaire [69]. Scores for the 8-item Patient Health Questionnaire range from 0 to 24, with higher scores indicating more severe depressive symptoms. The 7-item Generalized Anxiety Disorder scale [70] is used to assess symptoms of generalized anxiety, with scores ranging from 0 to 21 and higher scores indicating greater anxiety.

##### Other Assessments

Other assessments include the following: (1) putative mechanisms, (2) exploratory measures including NIH Helping to End Addiction Long-Term Initiative Core Data Elements measures, and (3) other auxiliary measures. These measures are included in the open pilot to test for feasibility of these assessments before the effectiveness-implementation trial.

##### Putative Mechanisms

Quota-based pacing is measured using a 4-item scale consisting of a subsection of items from the Activity Pacing Questionnaire [71]. Scores range from 5 to 20, with higher scores indicating increased pacing. The 13-item Pain Catastrophizing Scale [72] is used to assess pain rumination, magnification of the intensity of pain, and helplessness regarding the ability to cope with pain. Scores range from 0 to 52, with higher scores indicating increased pain catastrophizing. The Applied Mindfulness Process Scale [73] is used to assess the use of mindfulness in response to challenges of daily living. It consists of 15 items, and total scores range from 0 to 60, with higher scores indicating greater use of mindfulness. Similarly, participants’ broad conceptualization of mindfulness is assessed using the 12-item Cognitive and Affective Mindfulness Scale–Revised [74]. Its scores range from 12 to 48, with higher scores representing increased levels of mindfulness. We assess self-reported social support using the Interpersonal Support Evaluation List [75]. This measure consists of 12 items, and scores range from 12 to 48, with higher scores indicating strong social support. In contrast, the University of California Los Angeles–3 Loneliness Scale [76] is used to assess social isolation and perceived lack of social support. Total scores on this scale range from 3 to 9, with higher scores indicating greater social isolation. The participants’ ability to practice healthy coping skills is assessed using the Measure of Current Status [77]. Total scores on the Measure of Current Status range from 0 to 52, with higher scores reflecting stronger ability to recognize and cope with stress. The Self-Compassion Scale–Short Form [78] is used to assess a participant’s level of self-compassion. Scores are calculated by averaging the responses to the 12-item measure and range from 1 to 5. Higher scores are associated with higher levels of self-compassion. Finally, fear of physical activity is assessed by the Tampa Scale for Kinesiophobia–11. Scores range from 11 to 44, with higher scores reflecting more severe kinesiophobia [79].

This study is funded by the NIH Helping to End Addiction Long-Term Initiative, which requires core data elements to ensure that data can be compiled and analyzed across studies. In accordance with the data collection standards, pain interference is measured using the Pain, Enjoyment of Life, and General Activity scale [80], which assesses pain intensity and related interference in the enjoyment of life and activities of daily living. Scores range from 0 to 10, with higher scores indicating more severe pain and related interference. The Patient Global Impression of Change [81] is used to assess changes in pain in response to treatment. Scores range from 0 to 6, with higher scores indicating worse outcomes. The Tobacco, Alcohol, Prescription Medications, and Other Substance [82] screening questionnaire is used to assess substance use. Each substance is scored individually on a single-item scale of use ranging from 0 to 4, with lower scores indicating more frequent use. Sleep duration per night is measured using a single item from the Pittsburgh Sleep Quality Index [83]. Sleep quality is assessed using the Patient-Reported Outcomes Measurement Information System–Sleep Disturbance scale. Scores range from 6 to 30, with greater scores indicating more sleep disturbance.

##### Other Auxiliary Measures

We included measures to assess the elevated rates of trauma, stress, and discrimination in this population. We are using the 6-item Post-Traumatic Checklist–Civilian Version [84] to assess current symptoms of posttraumatic stress disorder. Scores range from 6 to 30, with higher scores indicating high posttraumatic stress disorder symptoms. The 4-item Perceived Stress Scale [85] is used to measure participants’ self-perception about stress levels. Total scores for this scale range from 0 to 16, with higher scores representative of more severe stress. To assess another significant stressor for many in this patient population, experiences of discrimination are measured using the Everyday Discrimination Scale–Short [86]. Total scores for this scale range from 0 to 25, with higher scores indicating greater frequency of discriminatory experiences.

##### Clinical and Demographic Variables

We assess general, pain-related information using the author-constructed Pain, Medication, and Medical History questionnaire, which measures self-reported (1) type of pain, (2) pain location and intensity, (3) pain treatments, (4) pain medications, (5) cannabis use for pain, (6) medical comorbidities, and (7) mental health conditions and medication. We additionally obtain the following demographic variables: age and birth date, gender, biological sex, race, ethnicity, education level, employment status, marital status, household income, disability status, language fluency, people in the household, Rural-Urban Commuting Area code (ie, zip code), country of birth, country lived in before the age of 12 years, years lived in the United States, parents’ countries of origin, ethnic identity, and languages spoken at home. There is no summary score; each item is scored individually.

Finally, exit interviews assess participants’ perceptions about the treatment and study procedures. Topics include what was the most and least helpful about the intervention, any topics that were not addressed that could be included, perceptions about each of the mind-body skills, comfort with participating in a skills-based treatment as opposed to a support group, and barriers to and facilitators of engagement with the material. Perceptions about the providers, assessments, homework, and group format will also be evaluated. RDA is used to analyze the exit interview data to inform the remaining open pilot groups and the subsequent trial.

For our Spanish open pilot, we used validated Spanish versions of the abovementioned questionnaires when they were available. A native speaker translated the remaining study measures.

### Data Analysis

Feasibility-related open pilot study measures will be assessed using a priori determined benchmarks for feasibility, acceptability, and fidelity based on our previous studies and recommendations in the field (Table 2) [87,88]. Given the concerns regarding the use of open pilot studies to calculate effect sizes [89,90], sample size estimates and proposed analyses were determined with the primary goal of establishing feasibility, as opposed to powering for specific effects for the quantitative outcomes. Preliminary effectiveness of the secondary outcomes will be examined using simple 2-tailed *t* tests. Evidence of effectiveness will be determined if the minimal clinically important difference for the specific quantitative outcome is included within the 95% CI for the change score.

### Ethical Considerations

The MGH institutional review board approved all the study procedures and determined that phase 1 of the study was exempt from written informed consent (institutional review board number for phase 1: 2022P001691). All phase 2 procedures were approved by the institutional review board before data collection (institutional review board number for phase 2: 2023P000362).

## Results

We began enrolling participants for the qualitative phase in November 2022 and the open pilot phase in May 2023. We conducted 25 provider interviews and 3 focus groups and 8 interviews with 18 English-speaking patients. The average age of the providers was 48.56 (SD 11.66) years. Most providers were women (21/25, 84%), non-Latino (22/25, 88%), and White (15/25, 60%). On average, they had 18.84 (SD 11.15) years of experience in working with patients, and approximately half (11/25, 44%) reported that they received mental health training as part of their degree. For demographic information about the patient qualitative and open pilot samples, refer to Table 3. RDA was completed before the initiation of the open pilot to inform the manual and program adaptations. The open pilot phase is ongoing. Older adults who participated in the qualitative phase were also invited to participate in the open pilot, with 44% (8/18) enrolling in the open pilot. We completed 2 English-speaking focus groups (group 1: 8/20, 40% and group 2: 6/20, 30%), and one is ongoing (6/20, 30%). In the first group, 100% (8/8) of the participants attended all the sessions, with only 1 dropout across the 2 groups so far. We have also conducted 3 individual interviews with Spanish-speaking older adults and will continue to obtain qualitative assessments with Spanish-speaking participants. We plan to conduct 2 open pilot groups in Spanish.

**Table 3:** Demographics for the patient qualitative assessments and open pilot.

Characteristics		English-speaking participants (qualitative; n=18)	Spanish-speaking participants (qualitative; n=3)	Open pilot participants (n=20)
Age (years), mean (SD)		65.72 (7.46)	59.67 (4.51)	70.20 (9.44)
Sex (female), n (%)		12 (67)	2 (67)	16 (80)
Gender (women), n (%)		12 (67)	2 (67)	16 (80)
**Ethnicity, n (%)**				
	Hispanic or Latino	1 (6)	3 (100)	3 (15)
	Non-Hispanic or Latino	16 (89)	0 (0)	15 (75)
	Unknown or not reported	1 (6)	0 (0)	2 (10)
**Race^a^, n (%)**				
	American Indian or Alaska Native	0 (0)	0 (0)	0 (0)
	Asian	0 (0)	0 (0)	0 (0)
	Black or African American	3 (17)	0 (0)	3 (15)
	Native Hawaiian or Pacific Islander	0 (0)	0 (0)	1 (5)
	White	15 (83)	1 (33)	13 (65)
	Unknown, not reported, or prefer not to answer	0 (0)	2 (67)	3 (15)
**Education, n (%)**				
	Less than high school	0 (0)	0 (0)	0 (0)
	Some secondary school or high school	2 (11)	1 (33)	2 (10)
	Completed high school or secondary school	8 (44)	0 (0)	8 (40)
	Completed associate or technical degree	1 (6)	0 (0)	2 (10)
	Completed college or baccalaureate degree	7 (39)	1 (33)	5 (25)
	Doctoral or postgraduate education	0 (0)	0 (0)	3 (15)
	Prefer not to answer	0 (0)	1 (33)	0 (0)
**Employment status, n (%)**				
	Full-time employment	3 (17)	1 (33)	3 (15)
	Not employed	2 (11)	2 (67)	17 (85)
	Part-time employment	13 (72)	0 (0)	0 (0)
**Household income (US $), n (%)**				
	<10,000	0 (0)	0 (0)	1 (5)
	10,000-24,999	6 (33)	1 (33)	7 (35)
	25,000-34,999	1 (6)	0 (0)	4 (20)
	35,000-49,999	3 (17)	0 (0)	2 (10)
	50,000-74,999	1 (6)	1 (33)	1 (5)
	75,000-99,999	0 (0)	0 (0)	2 (10)
	100,000-149,999	0 (0)	0 (0)	0 (0)
	150,000-199,999	1 (6)	0 (0)	0 (0)
	≥200,000	0 (0)	0 (0)	0 (0)
	Prefer not to answer	6 (33)	1 (33)	3 (15)
**Language or languages spoken^a^, n (%)**				
	English	18 (100)	0 (0)	20 (100)
	Spanish	0 (0)	3 (100)	1 (5)
	Arabic	1 (6)	0 (0)	0 (0)
	Other	3 (17)	0 (0)	1 (5)
**Country of birth, n (%)**				
	United States	15 (83)	0 (0)	15 (75)
	Other (ie, Egypt, Russia, Costa Rica, Honduras, Guatemala, Puerto Rico, Malta, or Sudan)	3 (17)	3 (100)	5 (25)
**Mother’s country of origin, n (%)**				
	United States	13 (72)	0 (0)	13 (65)
	Other (ie, Egypt, Russia, Costa Rica, Guatemala, Puerto Rico, Honduras, Malta, Sudan, Ireland, Italy, or Canada)	5 (28)	3 (100)	7 (35)
**Father’s country of origin, n (%)**				
	United States	13 (72)	0 (0)	11 (55)
	Other (ie, Jordan, Belarus, Costa Rica, Guatemala, Puerto Rico, Honduras, Malta, Sudan, Italy, Philippines, Oman, or Ireland)	5 (28)	3 (100)	9 (45)

^a^Participants may select multiple options; therefore, total percentages might exceed 100%.

## Discussion

### Principal Findings

This paper describes the study protocol for the adaptation and pilot effectiveness testing of a mind-body activity intervention, *GetActive+*, delivered as part of shared medical visits to older adults with chronic pain in an underserved community clinic. Older adults with chronic pain in community settings often lack access to evidence-based psychosocial treatment options. Given that their pain experience may be affected by socioeconomic factors that may influence the generalizability of interventions, we used a sequential approach to refine *GetActive+* for this setting. First, we conducted focus groups and qualitative interviews with patients and providers to evaluate patient experiences with pain and socioeconomic impacts on the pain experience; we also solicited feedback about the intervention components and logistical barriers to and facilitators of success. Next, we are conducting an open pilot with exit interviews to assess the intervention’s feasibility and acceptability and evaluate the signals of improvement in key outcomes, including physical function. We are conducting ongoing English-speaking groups. We are also simultaneously conducting focus groups and qualitative interviews with Spanish-speaking older adults to inform the open pilot in Spanish, which will be conducted by a Spanish-speaking provider.

Chronic pain interventions need to be adaptable to be easily disseminated to the millions of older adults with chronic pain, most of whom receive their treatment in community settings as opposed to academic medical centers where treatment trials are typically conducted. Shared medical visits represent an important avenue for increasing the access to evidence-based pain management interventions [38,91]; however, existing interventions have shown limited effects on key pain outcomes, including pain interference [34,39], possibly owing to overemphasis on broad education rather than skill training [35,37]. *GetActive+* is unique in its emphasis on activity engagement and the use of mind-body skills to facilitate increases in activity. Furthermore, our program emphasizes the development of skill mastery and group cohesion by reserving one-third to half of each session for checking in and troubleshooting skill use.

This protocol demonstrates the importance of flexibility in adapting an intervention to a community setting. We used an observational approach to training that allowed a nurse practitioner to work side by side with a psychologist to learn to deliver the intervention. In turn, the nurse practitioner contributed to the development of the clinician manual and training protocol. We will use a similar approach to train a social worker for the open pilot focus groups in Spanish. We reduced the number of skills in the intervention to increase the opportunity for group discussion and interpersonal connection. We increased the psychoeducation about the impact of stressors on pain, including incorporation of discussions about how stress, trauma, and grief influence the experience of pain throughout the treatment. We changed the language in the manual to be less wordy and increased the number of experiential exercises.

Results of this trial will inform a subsequent hybrid, implementation-effectiveness trial (N=200) to evaluate the effects of *GetActive+* on physical function (self-report, performance based, and step count), depression, and anxiety. In preparation for the trial, we will continue to refine the intervention based on both the quantitative and qualitative findings from the open pilot and feedback from the clinicians delivering the intervention. In the long term, if *GetActive+* proves to be successfully implemented and effective in this community clinic, we will aim to disseminate it broadly. The goal is to have an intervention that can be taught by any health professional, including those who have the most regular access to and strongest relationships with patients in these settings, such as medical interpreters or community health workers. Provider resources are limited, and consequently, there needs to be flexibility in who can administer psychosocial treatment. Thus, we aim to have an intervention for which any health practitioner can be trained to successfully deliver the group. To facilitate wide dissemination, our final training protocol and clinician manual for *GetActive+* will include specific guidelines and strategies oriented to address the training needs based on the type of provider, previous training, and expertise. If successful, this program will offer the potential for wide dissemination of mind-body skills with a focus on increasing physical activity, a key predictor of physical, cognitive, and emotional functioning in older adults.

### Limitations

Although we are currently only conducting focus groups or interviews and open pilot groups in English and Spanish, we aim to include groups for Cambodian-speaking and Arabic-speaking patients in the future. We hope to improve on this limitation in the subsequent trial. Furthermore, this clinic, although ethnically diverse, lacks a substantial proportion of African American patients; thus, they are underrepresented in our groups compared with the general US population.

### Conclusions

We have iteratively adapted and are currently testing the *GetActive+* mind-body activity intervention for older adult patients with chronic pain in a community clinic setting using shared medical visits. By the end of this trial, we will have a finalized intervention and study procedures that will enable us to test implementation and effectiveness in this setting. This trial has the potential to address the chronic pain epidemic by informing the adaptation of psychosocial pain management programs for wide dissemination in community clinics broadly. Such studies are essential for reducing health disparities and ensuring that historically underserved older adults with chronic pain have access to evidence-based pain management programs.

## Abbreviations

6MWT: 6-minute walk test
HIPAA: Health Insurance Portability and Accountability Act
MGH: Massachusetts General Hospital
NIH: National Institutes of Health
RDA: rapid data analysis
REDCap: Research Electronic Data Capture
RHC: Revere HealthCare Center

## Data Availability

Once the trial is complete, data will be made available upon request.
